# Diastolic Dysfunction is Associated with Liver Fibrosis and Insulin Resistance in Alcohol Use Disorder

**DOI:** 10.1007/s11606-025-10106-7

**Published:** 2026-01-30

**Authors:** Anna Hernández-Rubio, Sara Ceballos, Xavier García-Calvo, Paola Zuluaga, Javier López-Ayerbe, Luca Faconti, Robert Muga, Daniel Fuster

**Affiliations:** 1 Department of Medicine, Universitat Autònoma de Barcelona, Barcelona, Spain; 2 Germans Trias i Pujol Research Institute (IGTP), Badalona, Spain; 3 Department of Internal Medicine, Addiction Unit, Hospital Universitari Germans Trias i Pujol, Badalona, Spain; 4 Department of Cardiology, Hospital Universitari Germans Trias i Pujol, Badalona, Spain; 5 School of Cardiovascular and Metabolic Medicine & Sciences, King’s College, London, UK

**Keywords:** alcohol use disorder, diastolic dysfunction, echocardiography, cardiometabolic risk, insulin resistance, liver fibrosis

## Abstract

**Background:**

Alcohol use disorder (AUD) is associated with many health consequences, including cardiomyopathy with impaired systolic function, liver disease, and metabolic disturbances.

**Objective:**

We aimed to study the main echocardiographic abnormalities in otherwise asymptomatic individuals with AUD admitted for inpatient treatment, and to assess their associations with advanced liver fibrosis and insulin resistance, including their interaction.

**Design:**

A cross-sectional study.

**Participants:**

Individuals with AUD admitted for inpatient treatment between 2019 and 2024 in a university hospital in Spain.

**Main measures:**

We ascertained demographic information, alcohol use characteristics, anthropometric measurements, blood test parameters, and echocardiography findings upon admission. We estimated liver fibrosis with the FIB-4 index and insulin resistance with the triglyceride (TG)/HDL ratio. We performed multivariate logistic regression analyses to assess the main associations between cardiac abnormalities, advanced liver fibrosis, and insulin resistance.

**Results:**

We included 145 individuals (28% women), with a median age of 53 years [Interquartile range (IQR) 48–58 years], daily alcohol consumption of 10.3 standard drink units (SDU) [IQR 6.8–16.30 SDU], and body mass index of 25.8 kg/m^2^ [IQR 22.9–30.0 kg/m^2^]. Among them, 33% had hypertension and 14% had diabetes. The most common echocardiographic abnormality was diastolic dysfunction, which was present in 96 patients (66%), while systolic impairment was detected only in 7 cases (5%). Advanced liver fibrosis (FIB-4 > 3.25) was more prevalent in those with diastolic dysfunction (*p*-value = 0.010). In the multivariate logistic regression analysis, advanced liver fibrosis and TG/HDL ratio ≥ 3 were independently associated with diastolic dysfunction, with an OR of 3.53 (95% confidence interval (CI)1.20–10.41) and 4.55 (95% CI 1.57–13.22), respectively. However, no interaction between them was found (*p*-value for interaction = 0.92).

**Conclusions:**

Diastolic dysfunction is a common finding in apparently asymptomatic individuals with AUD admitted for inpatient treatment, and it is independently associated with advanced liver fibrosis and insulin resistance.

**Supplementary Information:**

The online version contains supplementary material available at https://doi.org/10.1007/s11606-025-10106-7.

## INTRODUCTION

Alcohol use is highly prevalent as up to 33% of the world’s population is estimated to consume alcohol^1^. Alcohol use can lead to a range of health and social consequences, and it is associated with the development of more than 200 different diseases^1^. Among them, neoplasms, unintentional injuries, gastrointestinal, and cardiovascular diseases were the leading contributors to the estimated 2.6 million alcohol-attributable deaths in 2019^2^. Alcohol use disorder (AUD) has the highest impact on individuals and population health due to its long-term consequences^3^. Alcohol-associated liver disease (ALD) through various stages, including steatosis, progressive liver fibrosis, and cirrhosis, is the most common alcohol-associated medical consequence^4^.

Cardiovascular and metabolic diseases are also prevalent in AUD, including hypertension, coronary heart disease, atrial fibrillation, hyperuricemia, and metabolic syndrome, among others. Chronic heavy alcohol consumption can lead to reduced insulin sensitivity or resistance, impaired secretion of insulin leading to impaired glucose tolerance, which increases the risk for type 2 diabetes, metabolic syndrome, and cardiovascular disease^5,6^. Mechanisms mediating these disturbances in AUD can be oxidative stress and alteration of the appetite regulating peptides (ghrelin and leptin), among others^7^.

Furthermore, recent Mendelian randomization studies show that any alcohol consumption is associated with increased cardiovascular risk, with marked risk differences across levels of intake^8,9^.

Echocardiographic abnormalities in individuals with AUD have been studied previously, mainly focusing on the detection of alcohol-induced cardiomyopathy (ACM)^10^. ACM has been described traditionally as an acquired form of dilated cardiomyopathy with low left-ventricle ejection fraction, chamber dilatation, and progression to congestive heart failure^11,12^. However, information on echocardiographic abnormalities in AUD has been mainly focused on the study of ACM, and data on other echocardiographic abnormalities in this context is scarce.

On the other hand, liver disease is highly prevalent in individuals with AUD, and its association with cardiac dysfunction is relevant. Previously, it was thought that cardiac dysfunction in cirrhosis was due to the direct toxic effect of ethanol, but new research elucidated that it could also be due to advanced liver disease itself, irrespective of the etiology^13^. However, information on these associations focusing on individuals with AUD is lacking.

To address these gaps of knowledge, we aimed to describe cardiac ultrasound abnormalities in individuals admitted for medical management of withdrawal syndrome. As a secondary objective, we aimed to address the association of the main cardiac alteration found with liver fibrosis and insulin resistance.

## METHODS

### Study Population

We performed a cross-sectional study of individuals with an AUD diagnosis, admitted for medical management of alcohol withdrawal syndrome in a tertiary hospital in Badalona, Spain (Hospital Universitari Germans Trias i Pujol), from June 2019 to September 2024. AUD was diagnosed according to the Diagnostic and Statistical Manual of Mental Disorders, 5th edition^14^. Addiction specialists at the primary care level referred patients for hospital treatment if they were deemed ineligible for outpatient medical withdrawal management or presented a high risk for severe alcohol withdrawal and/or co-occurring severe medical conditions. Patients were hospitalized for an average of 7 days, during which they received treatment with benzodiazepines for the management of alcohol withdrawal syndrome, as well as group-B vitamins to treat potential associated hypovitaminosis and its consequences (i.e., Wernicke-Korsakoff syndrome). Additional information on the admission protocol has been published elsewhere^15^. On the third day of admission, a transthoracic echocardiography was performed as part of the screening of alcohol-associated organ damage in those who met at least one of the following criteria: (1) long-term heavy alcohol consumption (more than 10 years of more than 2 drinks/day in men, 1drink/day in women), (2) cardiovascular risk factors or disease (hypertension, diabetes mellitus, dyslipidemia, metabolic syndrome, obesity, coronary artery disease, ischemic stroke, heart failure, and peripheral artery disease), and (3) age older than 40 years (for its increased risk of cardiac disturbances).

### Variables

On the day of admission, patients underwent a thorough medical history and physical examination to collect data regarding demographic (age and sex), alcohol use characteristics (i.e., daily alcohol consumption, age at initiation of alcohol use, duration of AUD, and previous treatment of the disorder), other substance use (including urine analysis), medical comorbidity (i.e., type 2 diabetes mellitus, hypertension, and dyslipidemia) and prior cardiovascular disease (i.e., coronary artery disease, ischemic stroke, and peripheral vascular disease). Alcohol use quantification was quantified in grams and converted to Standard Drink Units (SDU), following the United States conversion factors (11.671 g in 1 SDU)^16^. Anthropometric data (height and weight) were also recorded. On the second day of admission, a fasting blood sample was collected for complete blood count and biochemistry (total cholesterol, HDL-cholesterol, triglycerides, aspartate aminotransferase, alanine aminotransferase,and creatinine, among others). Liver fibrosis was estimated with the fibrosis-4 (FIB-4) index, which is calculated as follows: FIB-4 = (age × AST)/(platelet count × √ALT). FIB-4 was categorized as absence of liver fibrosis (< 2), intermediate (2–3.25), and advanced fibrosis (> 3.25)^17,18^. Insulin resistance was estimated through the TG/HDL ratio, with a cut-off value of 3.0^19^. The TG/HDL ratio is a widely used surrogate marker of metabolic syndrome, insulin resistance, and atherogenic dyslipidemia, which has been found to be associated with a worse cardiometabolic profile and increased cardiovascular risk^20^.

#### Echocardiographic Variables

Transthoracic echocardiography was performed by trained cardiologists using a Phillips EPIC CVx ultrasound system (Konin-klijke Philips N.V., Amsterdam, The Netherlands), according to the recommendations of the American Society of Echocardiography and the European Association of Cardiovascular Imaging^21,22^. Standard M-mode and 2D imaging of the left ventricle were undertaken at rest. For the evaluation of left ventricular mass, values were obtained in the parasternal long axis view immediately below the level of the mitral valve leaflet tips. Left ventricular mass was calculated using the cube formula and indexed to body surface area to obtain left ventricular mass index (LVMi). Left ventricular hypertrophy was defined by increased LVMi > 115 g/m^2^ in men and > 95 g/m^2 ^in women^23^.

Left ventricular function was assessed through a 4-chamber ejection fraction (LVEF) using the Simpson method and formula [(end diastolic volume − end systolic volume)/end diastolic volume]^21^. Systolic dysfunction was defined as decreased LVEF < 50%. Right ventricular function was assessed through Tricuspid Annular Plane Systolic Excursion (TAPSE) using M-mode in the apical four-chamber view.

Diastolic function was assessed with Doppler transmitral flow pattern by means of a pulsed mode obtained by placing the sample volume at the tips of the mitral leaflets. Peak velocity of the transmitral flow in early diastole (*E*, cm/s) and early diastolic motion of the mitral annulus (*e*′ wave) were measured through tissue Doppler imaging at either septal or lateral mitral annulus (cm/s), and the *E*/*e*′ ratio was calculated to estimate left ventricular filling pressures. Tricuspid regurgitation velocity was measured with continuous-wave Doppler from the apical four-chamber view, and left atrial volume was measured in the apical four-chamber and two-chamber view and indexed to the body surface area. Then, diastolic dysfunction was defined by the presence of two or more of the following echocardiographic abnormalities^22^: (a) *E*/*e*′ ratio > 14, (b) *E*′ velocity (< 7 cm/s at the septal mitral annulus or < 10 cm/s lateral mitral annulus), (c) tricuspid regurgitation velocity > 2.8 m/s, and (d) left atrial volume index > 34 mL/m^2^. Additionally, in individuals without sinus arrhythmia, peak velocity of atrial contraction flow (*A*, cm/s) was obtained by analysis of three consecutive cycles, and the *E*/*A* ratio was calculated.

### Statistical Analysis

We performed descriptive analyses using the median and interquartile range (IQR) for continuous variables and relative frequencies for categorical variables.

We conducted a *t*-test or Mann Whitney *U *test to assess differences between medians, where appropriate, whilst we used the Chi-squared test for the difference in proportions of the categorical variables. We also performed logistic regression analyses, adjusting for potential confounders to assess the association between main echocardiographic findings and liver fibrosis. We assessed the effect modification of insulin resistance, including the interaction term in the multivariate model.

We used the Homer-Lemeshow and Likelihood test ratio (LTR) to assess the goodness of fit. In addition, we performed two sensitivity analyses with a different FIB-4 cut-off level and excluding patients with prior cardiovascular disease.

*p*-values < 0.05 were considered statistically significant. We performed the statistical analysis using the Stata software (version 17.0; College Station, Texas, USA).

## RESULTS

### Baseline Characteristics

Among the 207 individuals with AUD consecutively admitted for inpatient treatment, 145 met the inclusion criteria and were included in the study. Among them, forty participants (27.6%) were women, 105 were men (72.4%), and the median age at admission was 53 years [IQR 48–58 years] (Table 1). The median age when patients started alcohol consumption was 16 years [IQR 16–18 years], and the average daily alcohol intake at admission was 10.3 SDU [IQR 6.8–16.3 SDU]. A total of 114 (78.6%) used nicotine, 45 (31.0%) were overweight (BMI ≥ 25 and < 30), 40 (27.6%) were obese (BMI ≥ 30), 48 (33.1%) had hypertension, and 20 (13.8%) had type 2 diabetes mellitus. The TG/HDL ratio was ≥ 3 in 45 (31.0%) individuals. Prior cardiovascular disease was present in 8 individuals (5.5%). Renal function was impaired (eGFR < 90 mL/min) in 28 patients (19.3%). Advanced liver fibrosis (FIB-4 > 3.25) was present in 40 individuals (27.6%).

**Table 1:** Baseline Characteristics of 145 AUD Patients Admitted for Inpatient Treatment

	***N*** = 145 Median [IQR]
Demographic and substance use variables	
Age (years)	53 [48–58]
Women	40 (27.6)
Age at first alcohol use (years), *N* = 134	16 [16–18]
Amount of daily alcohol consumption at admission	
Units: standard drink units	10.3 [6.8–16.3]
Units: g/day	120 [80–190]
Duration of regular alcohol consumption (months), *N* = 129	240 [132–360]
Cocaine in urine, *n* (%), *N* = 142	41 (28.3)
Cannabis in urine, *n* (%), *N* = 142	31 (21.4)
Opioids in urine, *n* (%)	4 (2.8)
Current nicotine use, *n* (%), *N* = 143	114 (78.6)
Cardiometabolic variables	
BMI (kg/m^2^), *n* (%), *N* = 141	25.8 [22.9–30.0]
Hypertension, *n* (%),	48 (33.1)
Diabetes mellitus, *n* (%)	20 (13.8)
Cardiovascular disease^*^, *n* (%)	8 (5.5)
Blood parametres and liver fibrosis	
Glucose (mg/dL)	96 [88–109]
Glycosylated hemoglobin (%), *N* = 137	5.4 [5.1–5.7]
C-reactive protein (mg/L), *N* = 144	2.55 [1.2–7.8]
Total cholesterol (mg/dL)	191 [162–222]
HDL-cholesterol (mg/dL), *N* = 142	58.30 [46.2–73.7]
LDL-cholesterol (mg/dL), *N* = 141	100 [77–134]
Triglycerides (mg/dL)	116 [82–186]
TG/HDL ratio ≥ 3, *n* (%), *N* = 142	45 (31.0)
Renal function (eGFR < 90 mL/min), *n* (%)	28 (19.3)
FIB-4 values, *n* (%)	
< 2.0	81 (55.9)
2–3.25	24 (16.5)
> 3.25	40 (27.6)

*BMI* body mass index, *HDL* high-density lipoprotein, *LDL* low-density lipoprotein, *TG* triglycerides, *eGFR* estimated glomerular filtration rate, *FIB-4* fibrosis-4 index, *IQR* interquartile range

^*^Cardiovascular disease includes coronary artery disease, ischemic stroke, and/or peripheral vascular disease

### Echocardiographic Findings

Diastolic dysfunction was present in 96 cases (66.2%) and left atrium enlargement in 45 (31.0%). Left ventricle hypertrophy was detected in 33 cases (22.8%). Seven individuals (4.9%) had systolic dysfunction (LVEF < 50%), and 5 out of 7 had segmental wall motion abnormalities. Right ventricle TAPSE was decreased in 41 individuals (28.3%), and 3 out of 41 had significant right ventricular systolic dysfunction (TAPSE ≤ 1.7 cm). During the assessment, median baseline systolic blood pressure was 123 mmHg [IQR 111–139 mmHg], diastolic blood pressure was 80 mmHg [IQR 70–88 mmHg], and heart rate was 74 bpm [IQR 70–80 bpm]. Table 2 shows the echocardiographic parameters of the study population.

**Table 2:** Main Echocardiographic Parameters in 145 AUD Patients Admitted for Inpatient Treatment

	***N* = 145** **Median [IQR]**
Left ventricle ejection fraction (LVEF, %), *N* = 143	62.6 [60.0–66.9]
Left ventricle mass index (g/m^2^), *N* = 131	90 [74–108]
Left ventricle diameter (cm), *N* = 142	4.5 [4.2–5.0]
Septum thickness (cm), *N* = 142	1.1 [1.0–1.2]
Posterior left ventricle wall thickness (cm), *N* = 141	1.0 [0.9–1.1]
E wave (cm/s), *N* = 133	59.2 [50.0–76.0]
A wave (cm/s), *N* = 129	67 [56–83]
E/A ratio, *N* = 130	0.81 [0.7–1.2]
Left Atrial diameter (cm), *N* = 138	3.7 [3.3–4.1]
Aortic root diameter (cm), *N* = 128	3.3 [3.0–3.5]
TAPSE (cm), *N* = 129	2.1 [1.9–2.4]

*IQR* interquartile range, *TAPSE Tricuspid Annular Plane Systolic Excursion. *

### Diastolic Dysfunction

**Figure 1:**
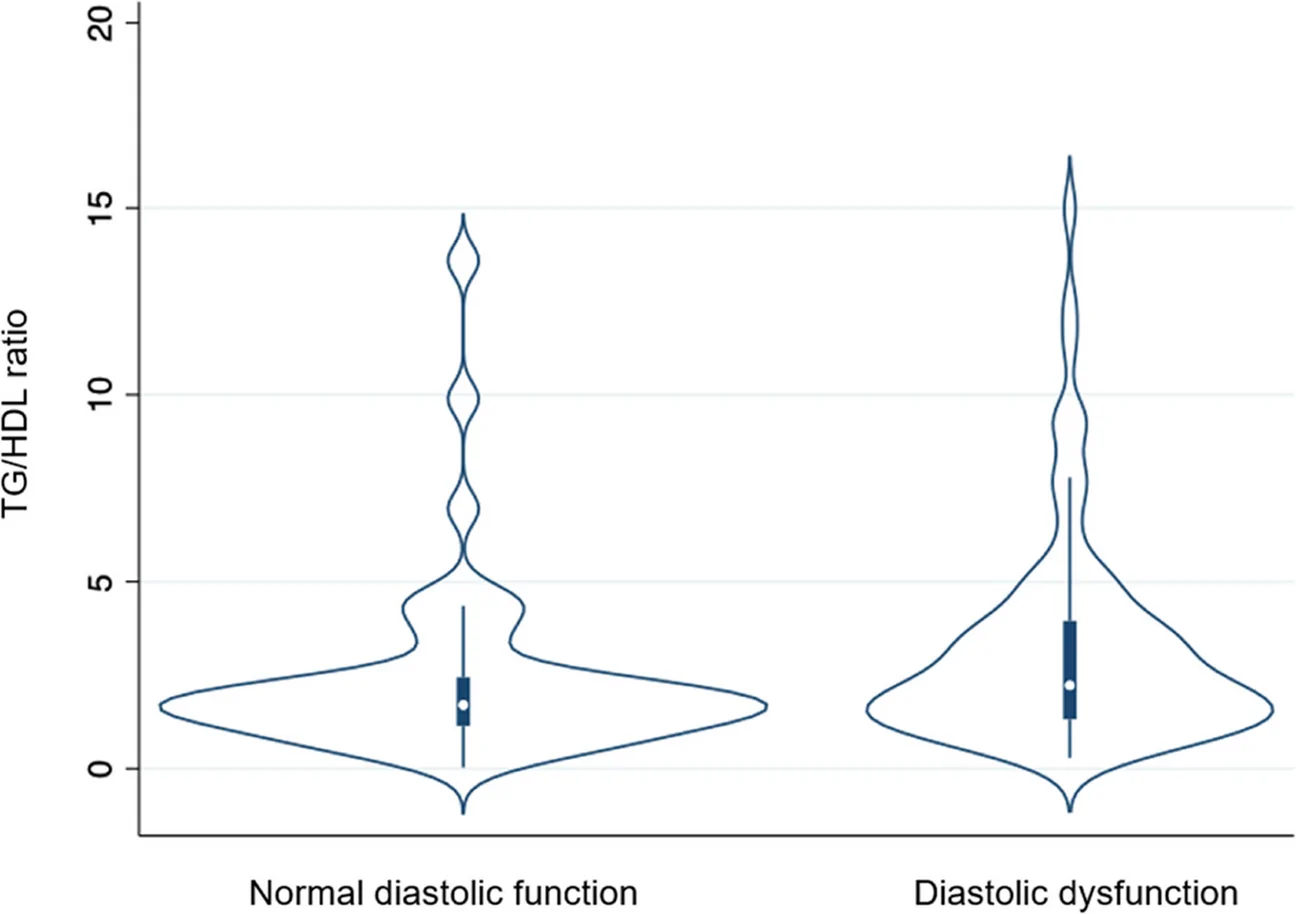
Violin plot for the distribution of TG/HDL ratio by diastolic dysfunction status. TG/HDL ratio, triglyceride-to-high-density lipoprotein cholesterol ratio

Table 3 shows the characteristics of patients with and without diastolic dysfunction. Noteworthy, individuals with diastolic dysfunction were older (*p* < 0.001), had a higher prevalence of hypertension (*p* = 0.051), decreased HDL-cholesterol (*p* = 0.042), increased TG/HDL index (*p* = 0.038), and advanced liver fibrosis (*p* = 0.010). Figure 1 shows the TG/HDL distribution in those with and without diastolic dysfunction.

**Table 3:** Characteristics of AUD Patients With and Without Diastolic Dysfunction

	Diastolic dysfunction, ***N*** = 96 ***Median [IQR]***	No diastolic dysfunction, ***N*** = 49 ***Median [IQR]***	***p***-value
Demographic and substance use variables			
Age (years)	55 [52–59]	49 [44–54]	< 0.01
Women	22 (22.9)	18 (36.7)	0.08
Age at first alcohol use (years old), *N* = 134	16 [15–18]	16 [16–18]	0.62
Amount of daily alcohol consumption (g/day)			
Units: standard drink units	10.3 [6.7–17.1]	10.3 [7.7–15.9]	0.76
Units: g/day	120 [80–200]	120 [90–185]	
Duration of regular alcohol consumption (months), *N* = 129	240 [250–384]	200 [120–360]	0.095
Cocaine in urine, *n* (%), *N* = 142	22(44.9)	33(34.4)	0.217
Cannabis in urine, *n* (%), *N* = 142	17(34.7)	24(25.0)	0.245
Opioids in urine, *n* (%)	3(6.1)	1(1.0)	0.101
Current nicotine use, *n* (%), *N* = 143	72 (75.0)	42 (85.7)	0.26
Cardiometabolic variables			
BMI (kg/m^2^), *N* = 141	26.0 [23.4–30.9]	25.29 [21.8–28.3]	0.092
Hypertension, *n* (%)	37 (38.5)	11 (22.4)	0.05
Diabetes mellitus, *n* (%)	16 (16.7)	4 (8.2)	0.16
Cardiovascular disease*, *n* (%)	5 (5.2)	3 (6.1)	0.82
Blood parametres and liver fibrosis			
Glucose (mg/dL)	98 [88–113]	93 [88–102]	0.07
Glycosylated hemoglobin (%), *N* = 137	5.4 [5.1–5.85]	5.3 [5–5.6]	0.15
C-Reactive Protein (mg/L), *N* = 144	2.7 [1.4–8.6]	2.2 [1.2–6]	0.47
Total Cholesterol (mg/dL)	192 [164–233]	190 [161–220]	0.57
HDL-Colesterol (mg/dL), *N* = 142	57 [43–73.7]	66.9 [49.3–74.6]	0.04
LDL-Colesterol (mg/dL), *N* = 141	101 [77–142]	99 [78–117]	0.39
Triglycerides (mg/dL)	125 [81–193.5]	106 [82–162]	0.23
TG/HDL ratio ≥ 3, *n* (%), *N* = 142	36 (37.5)	9 (18.4)	0.04
Renal function (eGFR < 90 mL/min), *n* (%)	78 (81.3)	39 (79.5)	0.81
FIB-4 values, *n* (%)			
< 2.0	45 (46.9)	36 (73.5)	0.01
2–3.25	19 (19.8)	5 (10.2)	
> 3.25	32 (33.3)	8 (16.3)	

*BMI* body mass index, *HDL* high-density lipoprotein, *LDL* low-density lipoprotein, *TG* triglycerides, *eGFR* estimated glomerular filtration rate, *FIB-4* fibrosis-4 index, *IQR* interquartile range

^*^Cardiovascular disease includes coronary artery disease, ischemic stroke, and/or peripheral vascular disease

### Multivariate Logistic Regression Analysis

We assessed the association between diastolic dysfunction and advanced liver fibrosis, adjusting for potential confounders (i.e., age, sex, hypertension, BMI, and TG/HDL). Table 4 shows that individuals with FIB-4 > 3.25 had increased odds of diastolic dysfunction (OR = 3.53, 95% CI 1.20–10.41, *p* = 0.022). Furthermore, TG/HDL ratio ≥ 3 was independently associated with diastolic dysfunction (OR = 4.55 (95% CI 1.57–13.22, *p* = 0.005), as well as age (13% increase for each 1-year unit, *p* < 0.001). The Homer-Lemeshow test resulted in a good model fitting, and LTR showed that the model fitting improved by adding the selected covariates (compared with the unadjusted model). When adding the interaction term between FIB-4 and TG/HDL, it resulted non-significant (*p* = 0.923, Supplementary Table S1).

**Table 4:** Multivariate Logistic Regression for the Associations of Diastolic Dysfunction in AUD Patients Admitted for Inpatient Treatment

	Crude OR (95% CI)	***p***-value	Adjusted OR (95% CI)	***p***-value
FIB-4 values*				
2–3.25	3.04 (1.03–8.94)	0.043	2.00 (0.59–6.74)	0.27
> 3.25	3.2 (1.31–7.79)	0.010	**3.53 (1.20–10.41)**	**0.02**
Hypertension	2.17 (0.99–4.76)	0.054	1.10 (0.42–2.82)	0.851
TG/HDL ratio ≥ 3	2.58 (1.12–5.95)	0.027	**4.55 (1.57–13.22)**	**0.01**
BMI**	1.07 (0.99–1.15)	0.078	1.03 (0.95–1.13)	0.44
Age**	1.11 (1.05–1.16)	< 0.001	**1.13 (1.06–1.20)**	**< 0.01**
Women	0.51 (0.24–1.08)	0.081	0.74 (0.28–1.95)	0.55

*CI* confidence interval, *FIB-4* fibrosis-4 index, *TG/HDL ratio* triglyceride-to-high-density lipoprotein cholesterol ratio, *BMI* body mass index, *OR* odds ratio

^*^Compared with FIB-4 < 2

^**^Age and BMI as continuous variables

Sensitivity analyses with a lower FIB-4 cut-off value and excluding individuals with previous cardiovascular disease showed results that were consistent with the primary model (Supplementary Table S2 and S3).

## DISCUSSION

Echocardiographic findings were common in this cross-sectional study of patients seeking treatment of AUD that are otherwise asymptomatic for cardiovascular disease. Diastolic dysfunction was observed in approximately two thirds of the cases and left ventricular hypertrophy in 23% in this case-series of individuals with AUD with a long duration of the disorder. Only a minority of the study population (less than 10%) had findings suggestive of systolic impairment.

Prevalence of diastolic dysfunction in the general population has been estimated to be around 27%, including 40% of individuals having a diagnosis of hypertension^24^. Diastolic dysfunction is thought to be an early sign of potential progression to heart failure with preserved ejection fraction. Previous literature on diastolic dysfunction in AUD patients is limited, with three studies showing impaired relaxation of the left ventricle and decreased *E*/*A *ratio in AUD^12,25,26^. However, echocardiographic methods in the past decades have changed towards more precise techniques such as tissue Doppler imaging for diastolic dysfunction assessment^27^, which makes it difficult to compare current and previous studies.

Additional evidence, although not specifically focused on individuals with AUD, has examined the association between alcohol consumption and echocardiographic abnormalities. A population-based study including 49,714 Korean adults who were screened for diastolic function and alcohol use revealed a dose-dependent association between alcohol use and diastolic dysfunction^28^, similar to other studies^29^.

Despite the prior literature connecting AUD with dilated cardiomyopathy and reduced systolic function, systolic impairment (LVEF < 50%) was an uncommon finding in our population. Furthermore, five out of the seven patients with systolic dysfunction had wall motion abnormalities, suggesting that aside from alcohol-induced cardiomyopathy, other etiologies (such as coronary heart disease) should be considered. Previous evidence on the prevalence of ACM among individuals with unexplained dilated cardiomyopathy has been reported to range from 3.8 to 47%^30,31^. However, background information is based on old papers that included few indicators of coronary artery disease, and comorbid or decompensated patients with other risk factors contributing to systolic dysfunction. Factors involved in the pathogenesis of ACM include myocardial damage through acetaldehyde (a toxic metabolite from alcohol), oxidative stress promoted by ethanol, the co-occurrence of cardiovascular risk factors, genetic susceptibility, and malnutrition. Additionally, ethanol has a direct toxic effect on cardiac myocytes, causing cell death, fibrosis, and impaired contractility, and activates the renin-angiotensin system, which contributes to progressive cardiac dysfunction^31^. These alterations can also lead to structural changes such as myocytes’ hypertrophy to avoid contractile depression in the early stages, which can lead to ventricular wall hypertrophy and compensatory dilatation in the later stages, leading to systolic impairment.^25,32,33^

Apart from alcohol use, 28% of our study population screened positive for cocaine use at admission (Table 1). This raises concern regarding the potential interaction between alcohol use and cocaine use over cardiac function, although no relevant differences in cocaine use were found between individuals with and without diastolic dysfunction (Table 3), remaining an area for future research.

Advanced liver fibrosis has been associated with both systolic and diastolic dysfunction through systemic inflammation, nitric oxide-induced vasodilatation, hemodynamic changes, and neurohormonal alterations^34^. The association between diastolic dysfunction and advanced liver disease has been studied previously in other populations^35^. In the context of metabolic-dysfunction associated steatotic liver disease), diastolic dysfunction is common due to the co-occurrence of metabolic syndrome, which increases the risk^36^. However, in ALD, the evidence is mainly in the setting of individuals with liver cirrhosis^37^. In this regard, the concept of cirrhotic cardiomyopathy encompasses subtle changes in cardiac function, evident by hyperdynamic circulation, combined with suboptimal contractile reserve and diastolic dysfunction, which is exclusive to individuals with cirrhosis. A recent study on 483 individuals with advanced ALD who were on the waiting list for liver transplant showed no cases of ACM (lowest ejection fraction of 55%), and 12.4% of individuals with diastolic dysfunction^38^. The discrepancy between this lower prevalence of diastolic dysfunction and the one found in our study (66%) could be due to differences in the study population; we have included otherwise asymptomatic individuals with AUD, where we screen for liver fibrosis and echocardiographic disturbances, not individuals with liver cirrhosis pending liver transplant (which may have fewer comorbidities that could be associated with diastolic dysfunction). In contrast, the prevalence of systolic dysfunction was more consistent between the two studies. Despite evidence linking ALD with diastolic dysfunction, whether ethanol through its main toxic metabolite, acetaldehyde, plays a synergistic role with liver fibrosis towards diastolic dysfunction remains to be elucidated.

Insulin resistance, measured through TG/HDL ratio, was found to be independently associated with diastolic dysfunction in the adjusted multivariate logistic regression analysis, increasing the odds after controlling for confounders (OR 4.55, 95% CI 1.57–13.22, *p* = 0.005), acting as a negative confounder. However, we did not detect interaction between TG/HDL and advanced liver fibrosis on the odds of diastolic dysfunction (*p*-value for interaction = 0.923, Supplementary Table S1). The TG/HDL ratio is considered a surrogate of both insulin resistance and atherogenic dyslipidemia, and has been associated with the metabolic syndrome and cardiovascular disease^20,39^. Recently, cardiometabolic disturbances and alcohol use have been found to have a synergistic role in organ damage, such as on liver disease^6,40^. Limited evidence to date has specifically addressed this synergy in the context of cardiac dysfunction.

The association between insulin resistance and diastolic dysfunction has been studied previously. In a population-based study that included 1063 individuals, the Homeostasis Model Assessment of Insulin Resistance correlated with *E*’ velocity and *E*/*E*’ ratio as markers of diastolic dysfunction^41^.

Other relevant associations from the multivariate analysis include age, which is strongly associated with diastolic dysfunction, increasing the odds 13% for each year increase. Age is associated with cardiac remodeling^42^and has previously been found to influence diastolic dysfunction^43^.

This study has several limitations that must be mentioned. First, information on lifestyle, diet, physical activity, and liver biopsy (gold standard for fibrosis detection) is lacking. Second, serum insulin measurement was lacking for detailed insulin resistance assessment, so the surrogate TG/HDL ratio was used. Third, individuals were admitted for medical management of withdrawal syndrome, so a hyperadrenergic state could be present during the assessments and could have overestimated the presence of diastolic dysfunction. However, none of the patients included developed severe symptoms of withdrawal. Finally, generalisability of these findings is limited to individuals with AUD who are admitted for hospital treatment and may not reflect what happens in patients with milder forms of unhealthy alcohol use. There are also strengths that have to be noted, such as the systematic screening for alcohol-associated organ impairment, including extensive blood tests and the estimation of liver fibrosis. Also, the fact that AUD patients included are not decompensated offers a unique opportunity to detect subclinical alcohol-associated cardiovascular damage.

Diastolic dysfunction in individuals with AUD admitted for detoxification is prevalent and is associated with advanced liver fibrosis and increased TG/HDL ratio. Systematic screening and treatment of metabolic disturbances, as well as early identification of diastolic dysfunction in AUD, can be crucial to help prevent progressive myocardial dysfunction. Future research with longitudinal data on the evolution of cardiac dysfunction is needed to better understand risk factors for progression.

## Supplementary Information

Below is the link to the electronic supplementary material.

ESM 1 (DOCX 35.5 KB)

## Data Availability

Data collected for the study are available to interested investigators upon request.
