# Supplementary material for: Diastolic Dysfunction is Associated with Liver Fibrosis and Insulin Resistance in Alcohol Use Disorder
Source: J Gen Intern Med. 2026 Jan 30;41(14):4112–20. doi: 10.1007/s11606-025-10106-7 (PMC13728062; doi:10.1007/s11606-025-10106-7)
Supplement: Supplementary file 1 [file 11606_2025_10106_MOESM1_ESM.docx]

# SUPPLEMENTAL MATERIAL

# Diastolic dysfunction is associated with liver fibrosis and insulin resistance in heavy drinkers.

# AUTHORS: Anna Hernandez-Rubio, MD, PhD ^1, 2^; Sara Ceballos ^1, 2^; Xavier García-Calvo, MD ^2, 3^; Paola Zuluaga, MD PhD ^1, 2, 3^; Javier López-Ayerbe, MD, PhD ^4^; Luca Faconti, MD, PhD ^5^; Robert Muga, MD, PhD ^1, 2, 3^; Daniel Fuster, MD, PhD ^1, 2, 3^.

^1^ Department of Medicine, Universitat Autònoma de Barcelona, Barcelona, Spain.

^2^ Germans Trias i Pujol Research Institute (IGTP), Badalona, Spain.

^3^ Department of Internal Medicine, Addiction Unit, Hospital Universitari Germans Trias i Pujol, Badalona, Spain.

^4^ Department of Cardiology, Hospital Universitari Germans Trias i Pujol, Badalona, Spain.

^5^ School of Cardiovascular and Metabolic Medicine & Sciences, King’s College, London, United Kingdom.

**Corresponding author:**

Daniel Fuster, M.D. Ph.D.

Department of Medicine, Universitat Autònoma de Barcelona, Barcelona, Spain.

e-mail: [2803814@uab.cat](mailto:2803814@uab.cat); dfuster.germanstrias@gencat.cat

Ctra Canyet s/n

Badalona 08916 (Barcelona)

SPAIN

Tel: +34 934978914

## Supplemental Table S1. Effect modification assessment for Insulin Resistance and Liver Fibrosis on diastolic dysfunction.

|  | **Adjusted OR (95 % CI)** | **p-value** |
| --- | --- | --- |
| FIB-4 values*   - 2 - 3.25 - > 3.25 | 2.03 (0.51-8.05)  3.11 (0.99-9.66) | 0.32  0.05 |
| TG/HDL ratio ≥ 3 | 4.14 (1.26-13.57) | 0.02 |
| FIB-4 # TG/HDL | 0.87 (0.05-14.74) | 0.92 |
| Hypertension | 1.10 (0.43-2.83) | 0.85 |
| BMI** | 1.03 (0.95-1.12) | 0.46 |
| Age** | 1.13 (1.06-1.20) | <0.01 |
| Women | 0.75 (0.28-1.96) | 0.55 |

*Compared with FIB-4 <2.

** Age and BMI as continuous variables.

Abbreviations: CI, confidence interval; FIB-4, fibrosis-4 index; TG/HDL ratio, triglyceride-to-high-density lipoprotein cholesterol ratio; BMI, body mass index.

## Supplemental Table S2. Sensitivity analysis for the multivariate model with a FIB-4 cut-off value of 2.67.

|  | **Adjusted OR (95 % CI)** | **p-value** |
| --- | --- | --- |
| FIB-4 values*   - ≥ 2 - <2.67 - ≥ 2.67 | 2.25 (0.51-9.91)  3.06 (1.13-8.30) | 0.282  0.028 |
| TG/HDL ratio ≥ 3 | 4.44 (1.53-12.85) | 0.006 |
| Hypertension | 1.07 (0.42-2.77) | 0.881 |
| BMI** | 1.04 (0.95-1.13) | 0.412 |
| Age** | 1.13 (1.06-1.20) | <0.001 |
| Women | 0.75 (0.29-1.97) | 0.559 |

*Compared with FIB-4 <2.

** Age and BMI as continuous variables.

Abbreviations: CI, confidence interval; FIB-4, fibrosis-4 index; TG/HDL ratio, triglyceride-to-high-density lipoprotein cholesterol ratio; BMI, body mass index.

## Supplemental Table S3. Sensitivity analysis for the multivariate model excluding individuals with previous cardiovascular disease.

|  | **Adjusted OR (95 % CI)** | **p-value** |
| --- | --- | --- |
| FIB-4 values*   - ≥ 2 - <2.67 - ≥ 2.67 | 1.88 (0.54-6.63)  3.45 (1.14-10.47) | 0.324  0.029 |
| TG/HDL ratio ≥ 3 | 5.07 (1.62-15.84) | 0.005 |
| Hypertension | 1.07 (0.39-2.97) | 0.889 |
| BMI** | 1.05 (0.96-1.16) | 0.307 |
| Age** | 1.13 (1.06-1.21) | <0.001 |
| Women | 0.72 (0.27-1.96) | 0.526 |

*Compared with FIB-4 <2.

** Age and BMI as continuous variables.

Abbreviations: CI, confidence interval; FIB-4, fibrosis-4 index; TG/HDL ratio, triglyceride-to-high-density lipoprotein cholesterol ratio; BMI, body mass index.
